# Multi-Locus Sequence Typing of a Geographically and Temporally Diverse Sample of the Highly Clonal Human Pathogen *Bartonella quintana*

**DOI:** 10.1371/journal.pone.0009765

**Published:** 2010-03-19

**Authors:** Mardjan Arvand, Didier Raoult, Edward J. Feil

**Affiliations:** 1 Hesse State Health Office, Centre for Health Protection, Dillenburg, Germany; 2 Faculté de Médicine de Marseille, Unité des Rickettsies CNRS UMR 6020, IFR 48, Université de la Méditerranée, Marseille, France; 3 Department of Biology and Biochemistry, University of Bath, Bath, United Kingdom; St. Petersburg Pasteur Institute, Russian Federation

## Abstract

*Bartonella quintana* is a re-emerging pathogen and the causative agent of a variety of disease manifestations in humans including trench fever. Various typing methods have been developed for *B. quintana*, but these tend to be limited by poor resolution and, in the case of gel-based methods, a lack of portability. Multilocus sequence typing (MLST) has been used to study the molecular epidemiology of a large number of pathogens, including *B. henselae*, a close relative of *B. quintana*. We developed a MLST scheme for *B. quintana* based on the 7 MLST loci employed for *B. henselae* with two additional loci to cover underrepresented regions of the *B. quintana* chromosome. A total of 16 *B. quintana* isolates spanning over 60 years and three continents were characterized. Allelic variation was detected in five of the nine loci. Although only 8/4270 (0.002%) of the nucleotide sites examined were variable over all loci, these polymorphisms resolved the 16 isolates into seven sequence types (STs). We also demonstrate that MLST can be applied on uncultured isolates by direct PCR from cardiac valve tissue, and suggest this method presents a promising approach for epidemiological studies in this highly clonal organism. Phylogenetic and clustering analyses suggest that two of the seven STs form a distinct lineage within the population.

## Introduction

*Bartonella quintana* is a fastidious, slow-growing, Gram-negative bacterium associated with a broad spectrum of disease manifestations, including trench fever, endocarditis, and bacillary angiomatosis [1], [2], [3]. Humans are the major reservoir for *B. quintana* and the human body louse is the principal vector [4], [5]. Recently, *B. quintana* was isolated by culture from the blood of a cynomolgus monkey [6] and *B. quintana* DNA was detected in specimens collected from cats and dogs and cat fleas, suggesting that other species may also be sporadically infected by this agent [7], [8], [9]. *B. quintana* is closely related to *B. henselae*, the agent of cat scratch disease, which can also cause bacillary angiomatosis, relapsing fever and endocarditis [3], [10]. Genome sequencing has revealed extensive genome reduction in *B. quintana*, suggesting that *B. quintana* is a genomic derivative of the more generalist species *B. henselae* [11].

Previous studies on genetic heterogeneity of *B. quintana* isolates have been carried out with different DNA fingerprinting methods including PCR–RFLP typing of the ribosomal intergenic spacer region, REP-PCR, ERIC-PCR, and pulsed-field gel electrophoresis (PFGE) [12], [13], [14]. These studies generally failed to reveal sufficient discrimination for epidemiological studies, suggesting that *B. quintana* is a highly conserved species. More recently, Foucault et al. described a Multispacer Typing (MST) method based on the sequence of highly polymorphic spacer regions of *B. quintana* [15]. This approach resolved only five MST profiles among 81 isolates and DNA extracts studied, which were mainly recovered from bacteremic homeless patients in France.

Multi-locus sequence typing (MLST) is based on the nucleotide sequences of housekeeping genes, and is a robust, standardizable and portable methodology that can be used in epidemiological and evolutionary studies [16], [17]. The aim of the present study was to develop a MLST scheme for *B. quintana*, based on the same genetic loci as those used previously for *B. henselae* [18], [19], [20], [21], [22]. We validated this scheme against a diverse sample of isolates representing three continents, over 60 years, and differing disease manifestations. Isolation of *B. quintana* by culture is often hampered by the fastidious nature of the organism, the requirement for prolonged incubation periods, and previous administration of antimicrobial chemotherapy. Therefore, the diagnosis is usually based on detection of bacterial DNA by PCR and/or serology. We sought to address the difficulties in culturing this organism by performing PCR directly from DNA collected from the cardiac valve tissue of a patient with culture-negative *B. quintana* endocarditis [23]. Analysis of the resulting data provides further evidence as to the clonality and phylogeny of the natural *B. quintana* population.

## Results

### Diversity among alleles and sequence types

All 16 isolates and the HROEH DNA extract were successfully sequenced at all nine MLST loci, with the sequenced alleles ranging in size between 416–510-bp at different loci (Table 1). Five of the nine loci were variable, *atp*F, *fts*Z, *gro*EL, *nlp*D, and *rpo*B, whereas *bqt*R, *gap*, *glt*A, and *rib*E were invariant. The average sequence divergence between all pairwise allelic comparisons was low, with the highest level being 0.4% observed in the *atp*F, *gro*EL and *rpo*B locus, and no loci exhibited more than 3 alleles (Table 1). On average the data suggest that *B. quintana* is approximately 10% diverged from *B. henselae* at MLST genes (data not shown). The data resolved the 16 *B. quintana* isolates into 7 sequence types (STs), the most frequent being STs 1 and 2, represented by 5 and 6 isolates, respectively (Table 2). STs 3–7 were represented by single isolates. The polymorphisms observed within the *fts*Z, *gro*EL, and *rpo*B loci were consistent with previous studies [24], [25], [26]. The two copies of the sequenced Toulouse isolate included in this study (Freiburg and CIP 103739) displayed an identical allelic profile, which differed from the Toulouse strain/copy Uppsala deposited in Genbank (BX897700.1) by a single base change at both the *rpoB* and *nlp*D loci (corresponding to allele 2 at these loci in the current study, and allele 1 at these loci in the Uppsala copy).

**Table 1:** Characteristics of the nine loci evaluated for the *B. quintana* MLST scheme.

Locus	Length of analysed sequence (bp)	No. of alleles	No. of variable sites	% sequence diversity [range (mean)]	Allelic polymorphism by reference to BX897700.1 (allele 1)
*atp*F	495	2	2	0.4	2: C391858T, T391985C
*bqt*R	510	1	0	0	-
*fts*Z	416	2	1	0.2	2: C1036988G
*gap*	504	1	0	0	-
*glt*A	450	1	0	0	-
*gro*EL	466	3	2	0.2–0.4 (0.3)	2: A1277454G, C1277746T 3: C1277746T
*nlp*D	461	2	1	0.2	2: A585446G
*rib*E	458	1	0	0	-
*rpo*B	510	3	2	0.2–0.4 (0.3)	2: G847761A 3: G847761A, G847791A

**Table 2:** Allelic profiles and sequence types (ST) obtained for the 16 *B. quintana* isolates and the HROEH DNA extract.

Strains	*atp*F	*bqt*R	*fts*Z	*gap*	*glt*A	*gro*EL	*nlp*D	*rib*E	*rpo*B	ST
SH-Perm	1	1	1	1	1	1	1	1	1	1
Oklahoma	1	1	1	1	1	1	1	1	1	1
Jouhanneau	1	1	1	1	1	1	1	1	1	1
UR.BQ.MBA 263	1	1	1	1	1	1	1	1	1	1
UR.BQ.MNHP 295	1	1	1	1	1	1	1	1	1	1
JK-31	1	1	1	1	1	1	1	1	2	2
BQ2D-70	1	1	1	1	1	1	1	1	2	2
HROEH (DNA)	1	1	1	1	1	1	1	1	2	2
UR.BQ.TIE 326	1	1	1	1	1	1	1	1	2	2
UR.BQ.MTF 357	1	1	1	1	1	1	1	1	2	2
UR.BQ.MNHP 374	1	1	1	1	1	1	1	1	2	2
Toulouse (copy Freiburg and CIP 103739)	1	1	1	1	1	1	2	1	2	3
Munich	1	1	1	1	1	2	1	1	2	4
Fuller	1	1	1	1	1	2	2	1	3	5
UR.BQ.MTF 335	1	1	2	1	1	3	1	1	2	6
Adelaide 1300/002	2	1	2	1	1	3	1	1	2	7

### Stability of the allelic polymorphisms

In order to evaluate the stability of the polymorphisms, four *B. quintana* isolates from different countries that were assigned to different STs were subjected to serial in vitro passages. The subculture 20 isolates were subsequently subjected to MLST analysis. The allelic sequences and profiles obtained from the serially passages isolates were 100% consistent with the results obtained from the primary isolates (data not shown). In addition, the clonally related isolates JK-31 and BQ2-D70, which represent different copies of the same original isolate, and the two copies of the Toulouse isolate included in this study (Freiburg and CIP 103739), revealed an identical allelic profile, indicating a high stability of the allelic polymorphisms detected in this study.

### Geographical and temporal distribution of *B. quintana* genotypes

There is little evidence for concordance between the MLST data and geographical source. Isolates from the same location exhibited different genotypes; for example, of the five isolates recovered from Marseille between 2002 and 2006, two corresponded to ST1, two to ST2, and one to ST6. Furthermore isolates from quite different locations exhibited the same genotypes; for example, ST1 corresponded to isolates from Russia, USA and France. The data also point to a remarkable temporal stability of this species. Most strikingly, the isolate SH-PERM (ST1), which was isolated in Russia during or shortly after the second world war, was identical over all nine loci (4270 bp) to an isolate from the USA sampled in 1990 and to three isolates from France sampled in the 1990ies, 2002 and 2003.

### Clonal and phylogenetic analysis

**Figure 1:**
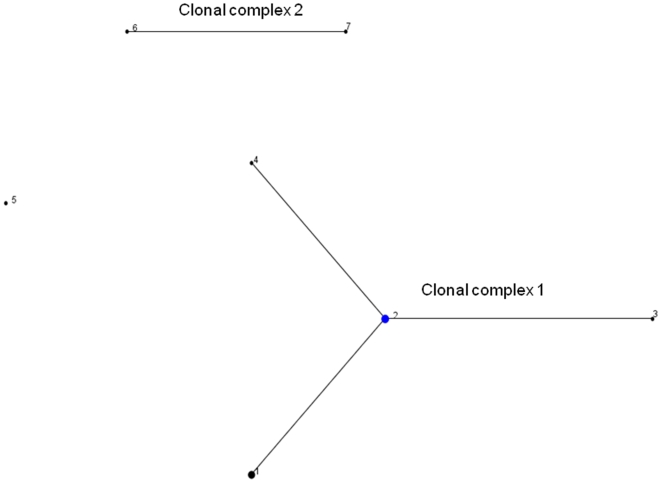
Phylogenetic relationship between different *B. quintana* STs as determined by eBURST. A clonal complex contains STs that have 8 out of 9 alleles in common. ST5 is assigned as a singleton since it differed in 3–7 alleles from all other STs. The size of the circles relates to the frequency of the corresponding ST, and illustrates that the assigned primary founder of the major clonal complex (ST2) is a common clone.

**Figure 2:**
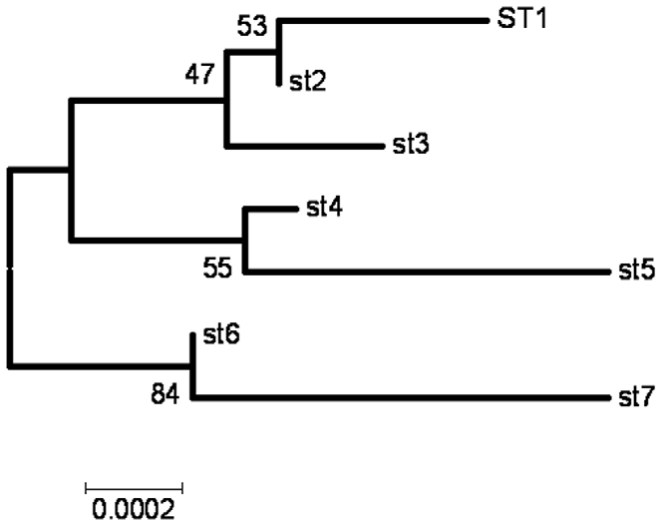
Neighbour-joining tree of the concatenated sequences of *B. quintana* STs as reconstructed by MEGA4. 1000 bootstrap replicates were used to examine the confidence in the tree. The clustering of STs 6 and 7 away from the other genotypes was supported by a bootstrap score of 84.

The relationships between the STs was first examined using eBURST, which uses allele profiles rather than sequences and does not attempt to reconstruct the relationships between the different clonal lineages. The majority of the isolates (13/16, 81.2%) corresponded to four STs, which formed a single clonal complex, clonal complex 1, with ST2 as primary founder, an assignment which is consistent with the fact this was the most commonly observed ST (Figure 1). STs 6 and 7 are single locus variants and ST5 is a singleton (differing at two or more genes from every other isolate). The clustering of STs 6 and 7 away from the other genotypes is also supported by neighbour-joining tree based on the concatenated sequences, with a bootstrap score of 84 (Figure 2).

## Discussion

Multi-locus sequence typing generates data which is highly discriminatory, reproducible, simple to perform, and portable, and here we describe a novel MLST scheme for *B. quintana*. The overall level of sequence divergence was very low, consistent with previous studies in this species. Four of the nine loci examined in this study were invariant, and future MLST studies on *B. quintana* might exclude these genes. Three of these invariant genes (*bat*R, *glt*A, and *rib*C) have previously been found to be polymorphic in *B. henselae* [18], [20], [22], indicating that *B. quintana* is more homogenous than *B. henselae*. This observation supports the view that the former derived from the latter. Despite the low level of variation in *B. quintana*, the 16 diverse isolates were resolved into 7 STs, which represents a favourable degree of discrimination relative to other typing schemes in this species. For example, PCR–RFLP, REP-PCR, ERIC-PCR, and PFGE have failed to reveal any variation between *B. quintana* isolates [12], [13], and MST resolved 5 genotypes among 71 *B. quintana* isolates and 10 DNA extracts studied [15]. It is important to note that the majority of isolates (51/71) examined in the latter study were obtained from the blood of bacteremic homeless people in Marseille. The overrepresentation of isolates from a specific group of patients from the same geographic region might explain the relative low level of sequence polymorphism detected among the *B. quintana* isolates examined by MST. In contrast, our isolate collection is, although smaller in size, a global panel containing isolates associated with different disease manifestations that were collected at different time points in 10 distinct geographic regions on three continents. Hence, it is not possible to compare the discriminatory power of MLST with MST on the basis of the results of our study. Further studies using the same panel of isolates are necessary to allow a formal comparison of both methods.

The stability and reproducibility of the MLST results were confirmed by subjecting four diverse isolates to serial in vitro passages and repeating the MLST analysis with 100% consistency. MLST also gave identical results for the JK-31 and BQ2-D70 isolates, which represent different copies of the same original isolate prior and after *in vivo* passages. We also demonstrate that MLST is possible via direct PCR from clinical samples, without the need to culture the isolate. The fastidious growth requirements of this organism means this presents a considerable advantage, although caution is urged to guard against misleading results arising from mixed infection, which may be evident as mixed peaks on the sequence chromatograms.

Although the two copies of the Toulouse strain included in this study (copy Freiburg and CIP 103739) produced identical results, these differed from the Toulouse strain/copy Uppsala, which has been deposited in GenBank [11]. Whilst it remains formally possible that this variation reflects changes during in vitro passage, we consider this unlikely due to the stability of this species both in the laboratory and in the natural population. A re-examination of the sequence data obtained by the whole genome sequencing, and/or the identity of the Toulouse strain/copy Uppsala should help to resolve this discrepancy.

The MLST data provide no evidence of geographical structuring and indicate striking clonal stability. In particular, ST1 corresponds to strains isolated approximately 60 years apart (SH-PERM and UR.BQ.MNHP 295). This temporal stability may reflect, in part, long generation times, consistent with intimate host association, genome degradation and the fastidious growth requirements of this organism in the laboratory. The lack of geographical structure suggests that rates of global migration, most likely via the human host, have been sufficiently high to disseminate the rarely emerging variants. It is noteworthy that this species is particularly associated with the two world wars of the last century, events which precipitated intense large-scale human migration. Further studies on a larger collection of isolates would help to improve our understanding of the temporal and geographic distribution of *B. quintana*.

Because it is based on the sequences of multiple housekeeping loci which are predominantly under stabilising selection, MLST data can also be used to examine the evolutionary relationships between strains [17]. Although the number of informative sites and allele diversity was too low for comprehensive analysis, eBURST and phylogenetic analysis revealed a consistent division between STs 6 and 7 and the rest of the *B. quintana* population. These genotypes correspond to isolates from France and Australia respectively, which again implies a lack of concordance between genetic variation and geographical source.

In summary, the *B. quintana* MLST scheme provides favourable levels of discrimination, within the context of a highly clonal species, and can be applied directly to clinical specimens containing uncultured isolates. The data provide evidence for a high level of temporal stability, consistent with long generation-times, and a robust phylogenetic division within the population. We note little evidence of geographic structuring, which implies high rates of global migration via the human host, although further studies on a larger strain collection are required to improve our understanding of the natural population of this highly clonal organism.

## Materials and Methods

### *B. quintana* isolates and growth conditions

Sixteen *B. quintana* isolates from six countries were analysed in this study, 14 of which were epidemiologically unrelated. Table 3 summarises the clinical and epidemiological data of all isolates studied. The BQ2-D70 isolate was isolated from the blood of a *Rhesus macaque* at day 70 after its experimental infection with JK-31 [27], hence BQ2-D70 represents JK-31 after multiple in vivo passages. The *B. quintana* Toulouse isolate, which has been recently sequenced [11], was included twice: as copy Freiburg (obtained from the Bartonella collection of the Institute for Medical Microbiology at the University of Freiburg, Germany [28], and CIP 103739 (obtained from the Culture Collection of the Institute Pasteur, Paris). The SH-Perm isolate was probably isolated in the city of Perm, Russia, from a patient suffering from trench fever during or after the second World War [10], [15]. HROEH was a DNA extract from the cardiac valve of a patient with culture-negative endocarditis by *B. quintana*, who underwent cardiac surgery in Rostock, Germany [23]. Strains were stored at −20°C or −80°C until use. The isolates were grown on Columbia agar with 5% sheep blood (Becton Dickenson) at 37°C in 5% CO_2_ for 7–14 d, and passaged once on blood agar prior to isolation of bacterial DNA.

**Table 3:** Characteristics of the 16 *B. quintana* isolates studied.

Isolate/DNA extract	Source of isolation, disease manifestation	Geographic origin	Isolation year	Reference
Fuller	Blood, trench fever	Yugoslavia	1945	[33]
SH-PERM	unknown, trench fever	Russia	World War II	[15]
Oklahoma	Blood, BA	Oklahoma city, USA	1990	[34]
JK-31	Tissue, BA	California, USA	Unknown	[27]
BQ2-D70	Blood, Rhesus macaque	California, USA	Unknown	[27]
Adelaide 1300/02	Blood, endocarditis	Adelaide, Australia	2002	[35]
Toulouse -copy Freiburg -CIP 103739	Blood, BA	Toulouse, France	1992	[36]
Jouhanneau	Blood, endocarditis	Paris, France	Unknown	this study
UR.BQ.MBA 263	Blood, BA	Marseille, France	2002	this study
UR.BQ.MNHP 295	Louse	Marseille, France	2003	this study
UR.BQ.TIE 326	Cardiac valve, endocarditis	Toulouse, France	2004	this study
UR.BQ.MTF 335	Blood, bacteraemia	Marseille, France	2005	this study
UR.BQ.MTF 357	Blood, bacteraemia	Marseille, France	2005	this study
UR.BQ.MNHP 374	Louse	Marseille, France	2006	this study
Munich	Blood, BA	Munich, Germany	1994	[37]
HROEH (DNA)	Cardiac valve, endocarditis	Rostock, Germany	2007	[23]

HROEH was a DNA extract from the cardiac valve tissue of a patient with culture-negative endocarditis by *B. quintana*.

1 BA, bacillary angiomatosis.

### Multi-locus sequence typing

**Figure 3:**
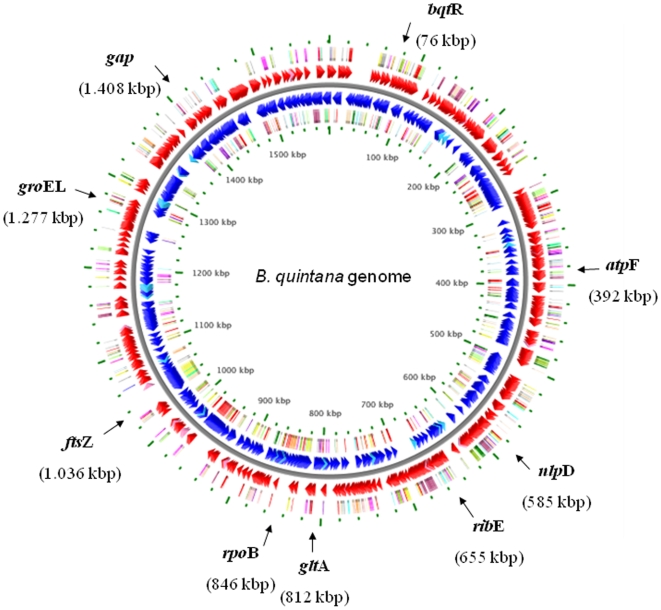
Localization of the 9 genetic loci used in the *B. quintana* MLST scheme on the chromosome of *B. quintana*. The genomic map was adapted from http://wishart.biology.ualberta.ca/BacMap/graphs_cgview.html [32]. The position of the loci has been marked by arrows.

Nucleotide sequence data were collected from 9 genetic loci presented in Table 1. Seven of these loci, i.e. *bqt*R (homologue to *bat*R), *fts*Z, *glt*A, *gro*EL, *nlp*D, *rib*E (homologue to *rib*C) and *rpo*B, are contained in the MLST scheme for *B. henselae* and were therefore selected for the MLST scheme for *B. quintana* [18], [22]. Two additional loci, *atp*F and *gap*, were selected because they are housekeeping genes that have been used in other MLST schemes [29], [30], and are located at underrepresented sites of the *B. quintana* chromosome. The distribution of alleles on the *B. quintana* chromosome is presented in Figure 3. Internal fragments of 531–622 length were amplified by using the primers presented in Table 4. PCR was performed in 50 µl volume as described previously [20]. Amplification was carried out for all loci by denaturation at 94°C for 5 min followed by 35 cycles (94°C for 1 min, 48°C for 30 s, and 72°C for 45 s) and a final extension step at 72°C for 6 min. The products were purified with a PCR purification kit from Quiagen Inc. (Hilden, Germany) and sequenced on both strands with the primers used for the initial amplification using the ABI PRISM BigDye Terminator cycle sequencing kit (Applied Biosystems) and a 3730XL DNA Analyzer (Applied Biosystems, Foster City, USA). The results were confirmed by repeats when necessary. The reliability of the sequence data was controlled by subjecting two randomly selected isolates in a blinded manner as “quality control strains” to the MLST analysis. The results of the quality control strains were 100% consistent with those obtained from the “original isolates”.

**Table 4:** Primers used for the amplification and sequencing of the nine genetic loci evaluated for the *B. quintana* MLST scheme.

Locus	Putative gene product	Product size (bp)	Position on chromosome (bp) 1	Forward primer (5′-3′)	Reverse primer (5′-3′)
*atpF*	ATP Synthase ß-chain	606	391.779–392.384	catcagagcatgcagatcgt	cgatgcacaatcatttctgg
*bqt*R	*B. quintana* transcriptional regulator	622	76.463–77.084	ttgcgacaaaacagcttcac	gatggagcatctgcactcaa
*fts*Z	cell devision protein ftsZ	531	1.036.753–1.037.283	ccatagcaaaagcatcagca	aattgaagccacgcattacc
*gap*	glyceraldehyde 3-phosphate dehydrogenase	612	1.408.521–1.409.132	caaaaccaatcccacagctt	ttgcgtgctattgtggagag
*glt*A	citrat synthase	558	811.826–812.383	gcggttatcctatcgaccaa	ctgctgcgatacatgcaaat
*gro*EL	Heat shock protein 60 chaperone	569	1.277.324–1.277.892	attggaggcattggagtgtc	cgcgtaaaccaaatcaaggt
*nlp*D	lipoprotein, outer membrane protein	548	584.964–585.511	gacgctttctcctgatggtc	tcaataaacgccctcgtacc
*rib*E	riboflavin synthase α-chain	575	654.876–655.450	gttcggaaatgaccgttgtt	gcttgtccccaagttgtcat
*rpo*B	RNA polymerase ß-subunit	605	847.962–847.358	tggtgatcgggtagagaagg	gacgcgcataaacattacga

1 Corresponding to the complete genome sequence of *B. quintana* strain Toulouse/copy Uppsala, GenBank accession number BX897700.1.

In one set of experiments, the *B. quintana* isolates Adelaide, Jouhanneau, JK-30 and BQ2-D70 were serially passaged on Columbia blood agar for 20 times. DNA isolated from the 20^th^ subculture was then subjected to MLST analysis again.

### Analysis of MLST Data

The nucleotide sequences were analysed with the DNASTAR Lasergene software package 7 (DNASTAR, Madison, USA). Different sequences of a given locus were given allele number, and each unique combination of alleles, i.e. the allelic profile, was assigned a sequence type (ST). The sequence of the *B. quintana* Toulouse/copy Uppsala (GenBank accession number BX897700.1) was assigned as allele 1 for each locus, and the allelic profile of this strain was assigned ST1.

### Phylogenetic analysis

The definition of clonal complexes and the examination of relationships between STs within clonal complexes were carried out by using eBURST (http://eburst.mlst.net). A neighbour-joining tree was reconstructed from the concatenated MLST alleles using the kimura-2-parameter distance measures as implemented in MEGA4 [31].

### Nucleotide sequence accession number

The sequences of the alleles from the *B. quintana* MLST scheme presented here have been deposited in GenBank under the following accession numbers GU946557 to GU946572.
